# Advanced low-thermal fortification strategy for dill juice: enhanced bioaccessibility and functional properties through MLP-RSM optimization

**DOI:** 10.3389/fnut.2025.1650490

**Published:** 2025-08-01

**Authors:** Seydi Yıkmış, Aylin Duman Altan, Selinay Demirel, Melikenur Türkol, Nazlı Tokatlı, Nazan Tokatlı Demirok, Moneera O. Aljobair, Emad Karrar, Isam A. Mohamed Ahmed

**Affiliations:** ^1^Department of Food Technology, Tekirdağ Namık Kemal University, Tekirdağ, Türkiye; ^2^Department of Industrial Engineering, Tekirdağ Namık Kemal University, Tekirdağ, Türkiye; ^3^Nutrition and Dietetics, Faculty of Health Sciences, Tekirdağ Namık Kemal University, Tekirdag, Türkiye; ^4^Department of Computer Engineering, Faculty of Engineering and Natural Sciences, Istanbul Health and Technology University, Istanbul, Türkiye; ^5^Department of Sports Health, College of Sports Sciences and Physical Activity, Princess Nourah Bint Abdulrahman University, Riyadh, Saudi Arabia; ^6^Department of Plant Sciences, North Dakota State University, Fargo, ND, United States; ^7^Department of Food Sciences and Nutrition, College of Food and Agricultural Sciences, King Saud University, Riyadh, Saudi Arabia

**Keywords:** *Anethum graveolens*, bioavailability, MLP optimization, non-thermal technology, ultrasound and microwave

## Abstract

In this study, a combination of ultrasound and microwave technologies (USMW) was applied to increase the functional properties of *Anethum graveolens* L. (dill) juice and the obtained samples were comprehensively evaluated in terms of biofunctionality. Total phenolic content (TPC), *β*-carotene, total chlorophyll, antioxidant capacity (FRAP) and antidiabetic enzyme inhibition (*α*-glucosidase, α-amylase) were determined. The optimum process parameters were successfully estimated by Response Surface Methodology (RSM) and Multilayer Perceptron (MLP) models. USMW process increased the extraction of phenolic compounds and carotenoids, providing significant increases in TPC (126.08 mg GAE/100 mL), *β*-carotene (42.82 mg/100 mL) and chlorophyll (4.42 g/100 mL) levels (*p < 0.05). In the simulated post-digestion bioavailability assessments, the ultrasound and microwave (DJ-USMW) group showed the highest recovery rates. In addition, potential antidiabetic effects were confirmed by the inhibition of *α*-glucosidase (61.65%) and α-amylase (53.11%). PCA and clustering analyses showed that USMW application significantly separated the samples. The obtained results demonstrate that USMW technology is a sustainable and effective method, especially for the development of functional beverages, as an alternative to traditional heat treatments.

## 1 Introduction

Plants and plant extracts attract attention due to their therapeutic and functional properties, as well as their richness in various bioactive components that are beneficial to health, their easy accessibility and affordability (1). Especially in recent years, functional beverages and products derived from aromatic plants have attracted increasing interest in the market. These plants are rich in health-supporting compounds such as phenolics, polyphenols, flavonoids, vitamins, minerals, amino acids, peptides (2–4). Thanks to their rich bioactive component content, they exhibit potential therapeutic effects on various chronic diseases and systems such as cancer, diabetes, cardiovascular diseases, intestinal health, immune system function and neurodegenerative diseases (5). In addition, secondary metabolites found in these plants are promising for human health as natural and safe pharmacological agents (6).

*Anethum graveolens* L., commonly known as dill, is an important aromatic, edible plant species belonging to the Apiaceae (Umbelliferae) family, originating from the Mediterranean and Western Asia (7, 8) This plant is extracted in the form of powder or oil from different parts including leaves, seeds and flowers, thus gaining various therapeutic values (6). Dill, which has a history dating back many years in traditional medicine, has a wide range of uses, including essential oil production, food additive, cosmetics, pharmaceuticals and the food industry (9). Dill contains volatile components with terpenoid structures such as limonene, carvone, dillapiol and estragole, as well as secondary metabolites such as flavonoids, tannins, coumarins, phenolic acids, terpenoids, fatty acids and water-soluble B group vitamins (B2, B5, B6, B7, B9), which reveals its striking profile and increases its bioactive potential (10, 11). This plant, which stands out with its antioxidant, antibacterial, antihyperlipidic and anti-inflammatory properties, attracts attention with its biological activities and is among the functional plant sources (12).

Due to consumer demand, interest in more environmentally friendly technologies that preserve product quality has increased, and in this context, the combination of microwaves and ultrasound reduces energy consumption and increases product quality by showing synergistic effects in food processing processes such as drying, frying, extraction and protein hydrolysis (13). While microwaves provide volumetric heating and shorten the processing time, ultrasound accelerates heat and mass transfer with the cavitation effect, so the cell structure of the food is broken down more easily, nutritional components are less deteriorated and energy consumption is reduced (14). Ultrasound and microwaves are becoming complementary technologies that offer various advantages in food processing. The combination of these technologies also shows its effectiveness in advanced processes such as dissolution, enzymatic hydrolysis and 3D food printing, allowing the development of more effective and sustainable food processing strategies. Therefore, the combined use of processes such as ultrasound and microwaves may be a good option (15).

In this study, the effects of ThermoMicrowave-Sonication (TMS) treatment applied to fresh dill juice on total phenolic content, bioactive compound profile, antioxidant capacity and potential bioavailability were evaluated. In this context, the potential of this innovative method involving minimal heat treatment to improve functional properties in sensitive plant materials such as dill has been demonstrated and an important gap in the existing literature has been filled. The results of the study aim to provide a scientific basis for the development of natural and sustainable food processing technologies.

## 2 Materials and methods

### 2.1 Preparation of dill juice

Dill (*Anethum graveolens* L.) samples were obtained from local agricultural producers in Tekirdağ region of Türkiye and stored under controlled conditions at +4°C to ensure biochemical stability during the pre-analysis process. During sample preparation, stems and mature tissues were removed. Mechanical homogenization was performed using a commercial blender of the Waring brand (Model HGB2WTS3), and particle size homogenization was ensured. The suspension was passed through a Whatman No. 1 filter paper to remove cellulosic residues, and then its macromolecular distribution was standardized with a vortex mixer (2000 rpm, 1 min). Untreated dill juice was called the control group (DJ-C).

### 2.2 Thermal pasteurization treatment

The dill juice samples were transferred to 100 mL glass bottles and pasteurized at 85 ± 1°C for 2 min using a water bath system (Wisd model WUC-D06H, Daihan, Wonju, Korea). Following pasteurisation, samples were cooled to room temperature and stored at −20 ± 1°C until further analysis thermal pasteurized (DJ-TP).

### 2.3 Ultrasound and microwave treatments

Samples of dill juice were subjected to different ultrasound parameters. The process was conducted on 100 mL of dill juice using a Hielscher Ultrasonics UP200St (Berlin, Germany). The frequency was 26 kHz, and the power was 200 W. Different amplitudes (60–100%) and treatment times (10–20 min) were used in continuous mode with an ice bath to prevent overheating. After ultrasonic treatment, the samples were cooled and stored at −18 ± 1°C. The application of ultrasound as a pretreatment prior to microwave processing has been shown to be more effective than its use following the microwave treatment (16). Since more effective results were obtained in preliminary analyses, microwave treatment was selected after ultrasound treatment in the study. Prior to treatment, fruit juice samples were equilibrated to room temperature. Subsequently, microwave irradiation was applied using a Samsung ME711K model (Kuala Lumpur, Malaysia) at power levels of 200, 450, and 700 W for durations of 20, 35, and 30 s, respectively.

### 2.4 Response surface methodology (RSM)

Dill juice was analyzed using the response surface method Minitab Statistical Analysis Software (Minitab 18.1.1) to understand the effect of ultrasound and microwave application on *β*-carotene and total chlorophyll parameters. There are 27 trial points for optimization (Table 1). The adequacy of the model was assessed by considering the *R*^2^ and the adjusted –*R*^2^ coefficients, the lack of fit tests, and the ANOVA results (Table 2). The Box–Behnken design was employed in this study. Independent variables were determined as ultrasound duration (min) (X_1_), ultrasound Amplitude (%) (X_2_), microwave duration (s) (X_3_), and microwave power (W) (X_4_). Dependent variables were selected, such as β-carotene and total chlorophyll values. The second-degree-polynomial equation shown in the following equation was used to create the model Equation 1:


(1) $$y=\beta_{0}+\sum_{i=1}^{3}\beta_{i}X_{i}+\sum_{i=1}^{3}\beta_{\mathrm{ii}}X_{i}^{2}+\sum_{\begin{array}{c} i=1\\ i<j \end{array}}^{3}\sum_{j=1}^{3}\beta_{\mathrm{ij}}X_{i}X_{j}$$

**Table 1:** Ultrasound and microwave RSM, MLP analysis of dependent and independent variables results.

Run no.	Independent variables				Dependent Variables					
	Ultrasound		Microwave							
					β-carotene (mg/100 mL)			Total Chlorophyll (g/100 mL)		
	Duration (X_1_) (min)	Amplitude (X_2_) (%)	Duration (X_3_) (s)	Power (X_4_) (W)						
					Experimental data	RSM predicted	MLP predicted	Experimental data	RSM predicted	MLP predicted
1	15	100	20	450	36.56	36.28	36.28	3.37	3.35	3.82
2	15	60	30	450	40.37	40.13	40.13	3.98	3.91	3.98
3	20	80	25	200	41.53	41.56	41.56	4.07	4.07	2.05
4	20	100	25	450	36.53	36.05	36.05	3.59	3.52	3.93
5	15	100	25	700	31.27	31.19	31.19	3.30	3.21	3.30
6	20	60	25	450	37.21	37.23	37.23	3.64	3.64	1.84
7	15	80	30	200	41.2	40.73	40.73	4.03	4.02	4.03
8	15	80	25	450	43.65	43.61	43.61	4.08	4.05	4.08
9	15	100	30	450	33.91	34.04	34.04	3.22	3.22	3.22
10	15	80	25	450	43.65	43.61	43.61	4.08	4.05	4.08
11	15	80	20	700	33.67	33.84	33.84	3.16	3.10	3.16
12	10	80	30	450	29.65	30.16	30.16	3.53	3.50	3.53
13	15	60	25	700	39.69	39.45	39.45	3.43	3.34	3.43
14	10	60	25	450	37.64	37.87	37.87	3.89	3.90	3.89
15	20	80	20	450	31.94	31.93	31.93	3.23	3.21	3.23
16	15	80	20	200	39.58	39.64	39.64	3.70	3.66	3.70
17	15	60	20	450	38.26	37.63	37.63	3.39	3.31	3.39
18	15	100	25	200	41.8	42.48	42.48	3.67	3.70	3.67
19	15	80	25	450	43.65	43.61	43.61	4.08	4.05	4.08
20	10	80	25	700	33.51	32.90	32.90	3.55	3.45	3.55
21	15	80	30	700	33.37	33.01	33.01	3.26	3.22	3.26
22	10	100	25	450	31.88	31.61	31.61	3.44	3.37	3.44
23	20	80	25	700	31.27	31.29	31.29	3.25	3.19	3.25
24	15	60	25	200	41.16	41.67	41.67	4.19	4.22	4.19
25	10	80	20	450	35.15	35.53	35.53	3.52	3.54	3.52
26	10	80	25	200	36.75	36.15	36.15	3.98	3.93	3.98
27	20	80	30	450	37.44	37.57	37.57	3.79	3.72	3.79
(RSM optimization parameters)	15.45	74.61	25.55	252.53	44.69			4.27		
Experimental values					42.85 ± 2.45			4.42 ± 0.14		
% Difference					4.11%			3.39%		
(MLP optimization parameters)	15.45	74.61	25.55	252.53	42.20			4.26		
Experimental values					42.85 ± 2.45			4.42 ± 0.14		
% Difference					1.52%			3.62%		

X_1_: Ultrasound duration (min); X_2_: Ultrasound amplitude (%); X_3_: Microwave duration (s); X_4_: Microwave power (W); RSM: Response surface methodology; MLP: Multi-Layer Perceptron.

**Table 2:** ANOVA in the regression model of the central combination test.

Source	DF	Total Chlorophyll (g/100 mL)		β-carotene (mg/100 mL)	
		*F*-value	*p*-value	*F*-value	*p*-value
Model	14.000	111.080	0.000	129.950	0.000
Linear	4.000	229.760	0.000	180.070	0.000
X_1_	1.000	5.440	0.038	42.670	0.000
X_2_	1.000	175.550	0.000	166.190	0.000
X_3_	1.000	97.350	0.000	0.200	0.664
X_4_	1.000	640.680	0.000	511.230	0.000
Square	4.000	111.300	0.000	198.900	0.000
X_1_X_1_	1.000	116.470	0.000	659.020	0.000
X_2_X_2_	1.000	176.070	0.000	117.040	0.000
X_3_X_3_	1.000	384.590	0.000	382.340	0.000
X_4_X_4_	1.000	97.940	0.000	136.190	0.000
2-Way Interaction	6.000	31.820	0.000	50.560	0.000
X_1_X_2_	1.000	22.160	0.001	25.730	0.000
X_1_X_3_	1.000	42.780	0.000	120.66	0.000
X_1_X_4_	1.000	21.790	0.001	49.080	0.000
X_2_X_3_	1.000	75.900	0.000	22.490	0.000
X_2_X_4_	1.000	20.900	0.001	81.750	0.000
X_3_X_4_	1.000	7.380	0.019	3.660	0.080
Error	12.000				
Lack-of-Fit	10.000	*	*	*	*
Pure Error	2.000				
Total	26.000				
*R*^2^		99.23%		99.34%	
Adj. *R*^2^		98.34%		98.58%	
Pred. *R*^2^		95.59%		96.23%	

X_1_: Ultrasound duration (min); X_2_: Ultrasound amplitude (%); X_3_: Microwave duration (s); X_4_: Microwave power (W); DF: degrees of freedom; *R*^2^—coefficient of determination; *p* < 0.05, significant differences; *p* < 0.01, very significant differences; *The pure error was zero; thus, the Lack-of-Fit *F*-value and *p*-value could not be computed. This indicates that the model fits the data without detectable deviation.

In the equation, Y is the dependent variable, βo is the intercept term, βi is the first-degree (linear) equation coefficient, βii is the second-degree equation coefficient, βij is the two-factor cross-interaction coefficient, and Xi and Xj are the independent variables.

### 2.5 Multilayer perceptron (MLP) neural network

**Figure 1:**
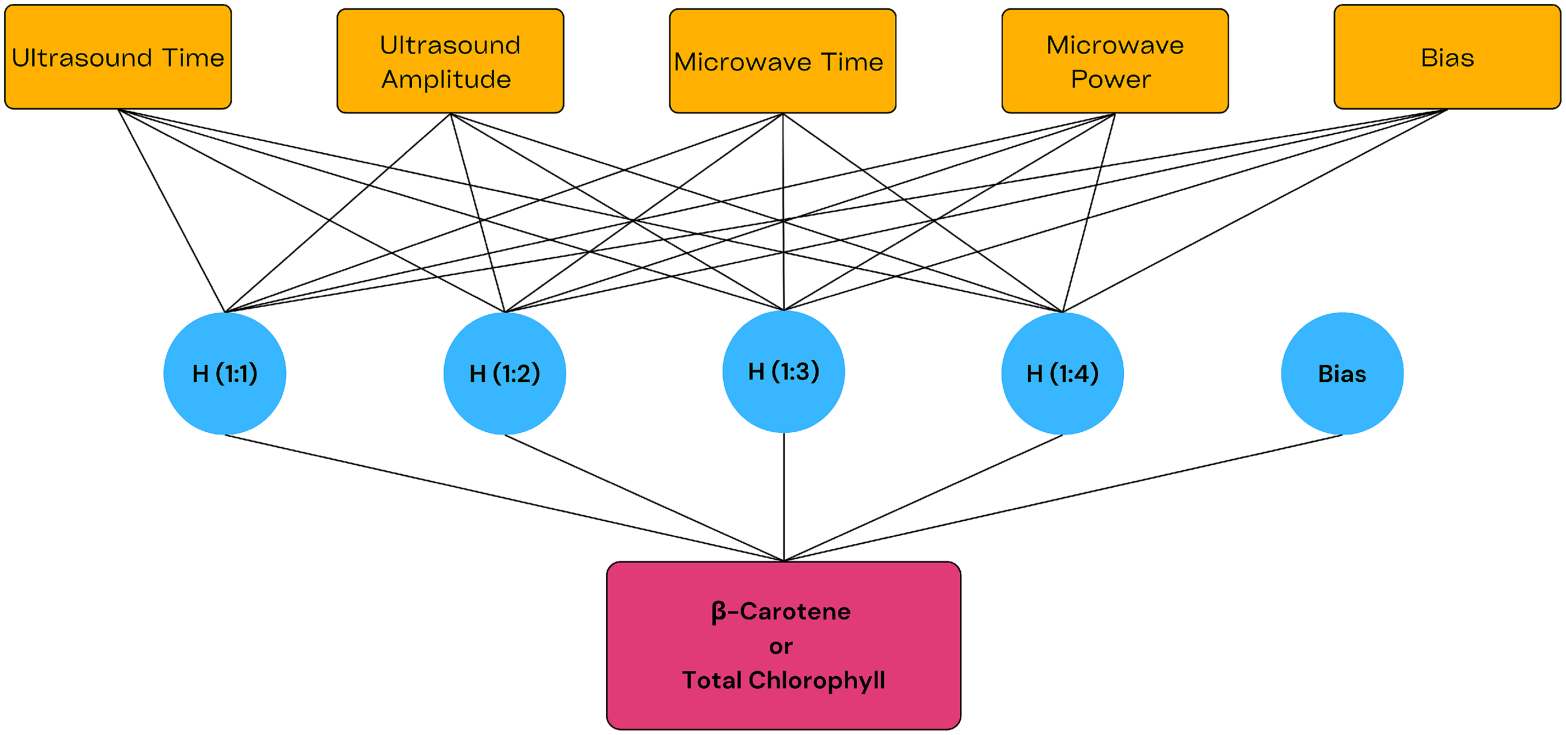
Structure of MLP model.

An MLP model consists of an input layer, at least one hidden layer and an output layer. In MLP, results are calculated using the weights and activation functions of the input data and this process is repeated until the output layer is reached (17). The relative predictive power was determined using normalized scores and a machine learning algorithm (MLP) sensitivity analysis. The neural network fitting tool of IBM SPSS Statistics 27 was employed for modeling of experimental data (with 56 samples). The model input variables consisted of Ultrasound duration (X_1_) (min), Ultrasound amplitude (X_2_) (%), Microwave duration (X_3_) (s) and Microwave power (X_4_) (W), the *β*-Carotene (mg/100 mL) or Total Chlorophyll (g/100 mL) was considered as the model output. The model structure, which uses tanh (hyperbolic tangent) in the hidden layer and identity activation functions in the output layer, is presented in Figure 1. In addition, the order of importance of variables in the sensitivity analysis results was used to analyze the relative effects of variables on the model. In this way, it was determined which input variable was more effective in predicting the output variable.

### 2.6 Determination of bioactive compounds

The total phenolic content was determined using the Folin–Ciocalteu method, with the results expressed as milligrams of gallic acid equivalent per liter (18). In order to determine the chlorophyll content in dill juice samples, the spectrophotometric method developed by Hiscox and Israelstam was used in the literature (19). During the analysis process, 3 mL of dill extract and 3 mL of acetone solution (80% v/v) were mixed homogeneously. The mixture was purified by passing through Whatman brand filter paper three times. In the last stage, the filtrate’s light absorption values (absorbance) were measured spectrophotometrically at 645 and 663 nanometers. The total antioxidant activity of the samples was determined using the FRAP (Ferric Reducing Antioxidant Power) test. The test principle is based on reducing Fe^3+^ to Fe^2+^, thus forming a colored complex. The absorbance value of this complex was measured spectrophotometrically at 593 nm. The standard curve was generated using Trolox (a synthetic antioxidant analog) and the results were reported as millimoles of Trolox equivalents (mmol TE/L) of antioxidant activity per liter of dill juice (20).

Total carotenoid content was determined using minor modifications of spectroscopic methods to analyze dill juice samples (21, 22). A 1 mL aliquot of dill juice was mixed with 5 mL methanol solution (1:2, v/v). The mixture was allowed to stand until phase separation occurred, after which the upper phase was carefully separated. 0.5 mL of saturated sodium chloride solution was added to this upper phase, and the mixture was shaken again. A small amount of sodium sulfate was added to the lower phase and the solution was centrifuged at 4000 rpm for 10 min. After centrifugation (GYROZEN, 1730 R, Korea) the upper phase was taken up again and 5 mL of methanol solution was added. The resulting mixture was analyzed with a UV–visible spectrophotometer (SP-UV/VIS-300SRB, Spectrum Instruments, Victoria, Australia) at a wavelength of 450 nm. The absorbance values were compared against a calibration curve generated from *β*-carotene standard solutions, and the total carotenoid content in the juice was determined as mg β-carotene equivalent per liter.

### 2.7 Identification of phenolic compounds

Phenolic compounds were analyzed using an Agilent 1,260 Infinity chromatograph with a diode array detector (DAD). As outlined in the study by Portu et al. (2016), the chromatography process was carried out utilizing a C-18 Agilent column (250 × 4.6 mm; 5 μm packing) (23). The column temperature was fixed at 30°C with a flow rate of 0.80 mL/min. Detection was carried out at 280, 320, and 360 nm. The phenolic compounds analyzed in the study included chlorogenic acid, catechin hydrate, 4-hydroxybenzoic acid, p-coumaric acid, t-ferulic acid, hydroxycinnamic acid, o-coumaric acid, quercetin, naringenin, and chrysin. Calibration curves for each phenolic acid were established in the concentration range of 2.5 to 250 mg/L. The validation curves obtained showed R^2^ values above 0.99 for all compounds, demonstrating high accuracy of measurement. The concentrations of these compounds are expressed as μg/mL. The results for phenolic compounds are given as the average of the analyses of three samples.

### 2.8 Antidiabetic activity

Using a modified process, the *α*-glucosidase and α-amylase inhibition activities were evaluated to determine dill juice’s potential as an antidiabetic (24). The positive control in these analyses was acarbose (0.2 mL). Dill juice samples (0–200 μL) were reacted with *α*-amylase at a concentration of 0.5 mg/mL in 0.02 M sodium phosphate buffer solution (500 μL, pH 6.9) at 25°C for 10 min. A UV–VIS spectrophotometer (Spectrum Instrument, SP-UV/VIS-300SRB, Australia) was used to measure absorbance at 540 nm for α-amylase inhibition activity. Dill juice samples (100 μL) were mixed with 100 μL of 0.1 mol/L phosphate buffer (pH 6.9) and 100 μL of α-glucosidase solution (1 U/mL) and incubated at 25°C for 5 min. Then, 100 μL of p-nitrophenyl-α-D-glucopyranoside (5 mmol/L) was added and the mixture was incubated at 25°C for 10 min. The absorbance was measured at 405 nm and α-glucosidase inhibition (%) was calculated.

### 2.9 *In vitro*-simulated gastrointestinal digestion analysis

An *in vitro* digestion model was used followed by dialysis according to the method of Minekus et al. (25). The methodology consists of three sequential phases, including the oral (α-amylase, pH 7.0), gastric (pepsin, pH 3.0), and intestinal (pancreatin and fresh bile, pH 7.0) phases. Digestions and determinations of total chlorophyll (g/100 mL), *β*-carotene (mg/100 mL), TPC (mg GAE/100 mL), and FRAP (mmol TE/L) were performed after the gastric and intestinal phases and were determined in triplicate for each treatment and replicate.

### 2.10 Statistical analysis

All experimental procedures were conducted in triplicate to ensure reproducibility. Results are presented as mean values accompanied by standard deviations (mean ± SD). Statistical evaluation of the data was carried out using one-way analysis of variance (ANOVA), and post-hoc comparisons among group means were performed using Tukey’s Honest Significant Difference (HSD) test, with statistical significance set at *p* < 0.05. All statistical tests were performed using SPSS software (version 22.0; SPSS Inc., Chicago, IL, USA). Three-dimensional response surface methodology (RSM) plots were created using SigmaPlot software (version 12.0; Systat Software, Inc., San Jose, CA, USA). Multiple linear regression (MLR) analyses were implemented in Python (version 3.9) via the Spyder integrated development environment (IDE, version 5.4.3) within the Anaconda distribution (Anaconda Inc., Austin, TX, USA).

## 3 Results and discussion

### 3.1 Evaluation of results obtained with RSM

In this study, the effects of the combined use of ultrasound and microwave technologies on the total chlorophyll and *β*-carotene content of dill juice were investigated. The influence of ultrasound parameters X_1_: Ultrasound duration (min) and X_2_: Ultrasound amplitude (%) and microwave parameters X_3_: Microwave duration (s) and X_4_: Microwave power (W) on the total chlorophyll (Equation 2) and *β*-carotene (Equation 3) contents of dill water are presented in the following equations.


(2) $$\begin{array}{l} \text{Total Chlorophyll}\;(g/100\;\mathrm{mL})=-11.177+0.0489\;X_{1}\\ +0.11111\;X_{2}+0.8243\;X_{3}+0.002152\;X_{4}\\ -0.007875\;X_{1}X_{1}-0.000605\;X_{2}X_{2}-0.014310\;X_{3}X_{3}\\ -0.000003\;X_{4}X_{4}+0.000992\;X_{1}X_{2}+0.005511\;X_{1}X_{3}\\ -0.000079\;X_{1}X_{4}-0.001835\;X_{2}X_{3}+0.000019\;X_{2}X_{4}\\ -0.000046\;X_{3}X_{4} \end{array}$$

(3) $$\begin{array}{l} \beta -\text{carotene}\;(\mathrm{mg}/100\;\mathrm{mL})=-145.4+3.736\;X_{1}+1.156\;X_{2}\\ +7.969\;X_{3}+0.09026\;X_{4}-0.22278\;X_{1}X_{1}-0.005868\;X_{2}X_{2}\\ -0.16969\;X_{3}X_{3}-0.000041\;X_{4}X_{4}+0.01271\;X_{1}X_{2}\\ +0.1101\;X_{1}X_{3}-0.001404\;X_{1}X_{4}-0.01188\;X_{2}X_{3}\\ -0.000453\;X_{2}X_{4}-0.000383\;X_{3}X_{4} \end{array}$$

According to the optimization results based on Response Surface Methodology (RSM), the independent variables (ultrasound duration, amplitude, microwave duration, and power) had significant effects on both bioactive compounds. In particular, microwave power (X_4_) and ultrasound amplitude (X_2_) exhibited the most pronounced effects on both total chlorophyll and *β*-carotene levels (*p* < 0.001). The high *R*^2^ values of the models (99.23% for total chlorophyll and 99.34% for β-carotene) indicate the strong predictive power of the applied model. Similarly, Zhang et al. (26) reported an increase in β-carotene levels following ultrasound treatment in their study on different squash juices, supporting the findings of the present study (26). In the study conducted by Suo et al. (27), a significant increase in carotenoid content was observed in ultrasound-treated squash juice compared to the untreated sample (27). Unlike single-factor experimental methods and some algorithms, RSM allows the process to be optimized in a multi-dimensional way by examining the effects of multiple independent variables on a dependent variable simultaneously. This approach shows that RSM has a mechanism that simultaneously evaluates all the factors that are effective in the process (28).

ANOVA analyses demonstrated that all components of the model (linear, quadratic, and interaction terms) were statistically significant. Among the variables, microwave power (X_4_) exhibited the highest *F*-value, with *F* = 640.68 for total chlorophyll and *F* = 511.23 for *β*-carotene. Additionally, interaction terms such as X_1_ × X_3_ and X_2_ × X_4_ were found to have synergistic effects on both response variables. These findings indicate that the process parameters produce significant outcomes not only through their individual effects but also through their interactions with one another.

Under the optimized conditions (15.45 min ultrasound duration, 74.61% amplitude, 25.55 s microwave duration, and 252.53 W power), the experimentally determined values were 42.85 ± 2.45 mg/100 mL for β-carotene and 4.42 ± 0.14 g/100 mL for total chlorophyll. These results showed strong agreement with the predicted values obtained from RSM, with percentage differences of 4.11 and 3.39%, respectively. This supports the validity and predictive accuracy of the model, indicating a high level of experimental reliability. The strong predictive power of RSM has also been confirmed in other similar studies (29–31). In a study by Zeb et al. (32), similar to our findings, microwave treatment was reported to enhance the carotenoid and chlorophyll contents of *Momordica charantia* L. (32).

In conclusion, the combined application of ultrasound and microwave treatments significantly enhanced the functional components of dill juice, demonstrating the synergistic effects of these technologies. The optimized conditions obtained through RSM offer potential for functional beverage formulation and industrial applications. This study suggests that the ultrasound–microwave combination may serve as a sustainable and effective bioactive enrichment strategy, providing a promising alternative to conventional thermal processing methods. In a study conducted on papaya, ultrasound-assisted extraction (MPUAE) with microwave pretreatment was compared with ultrasound-assisted extraction (UAE) alone and it was determined that MPUAE provided higher energy efficiency in obtaining bioactive compounds. The findings show that combined applications such as MPUAE offer a more environmentally sustainable alternative (33). A systematic review, on the other hand, points out that although ultrasound technology is a promising method in the field of modern food processing with its compliance with sustainability principles and functional potential, comprehensive interdisciplinary research needs to be continued in order for this potential to be fully utilized on an industrial scale (34).

**Figure 2:**
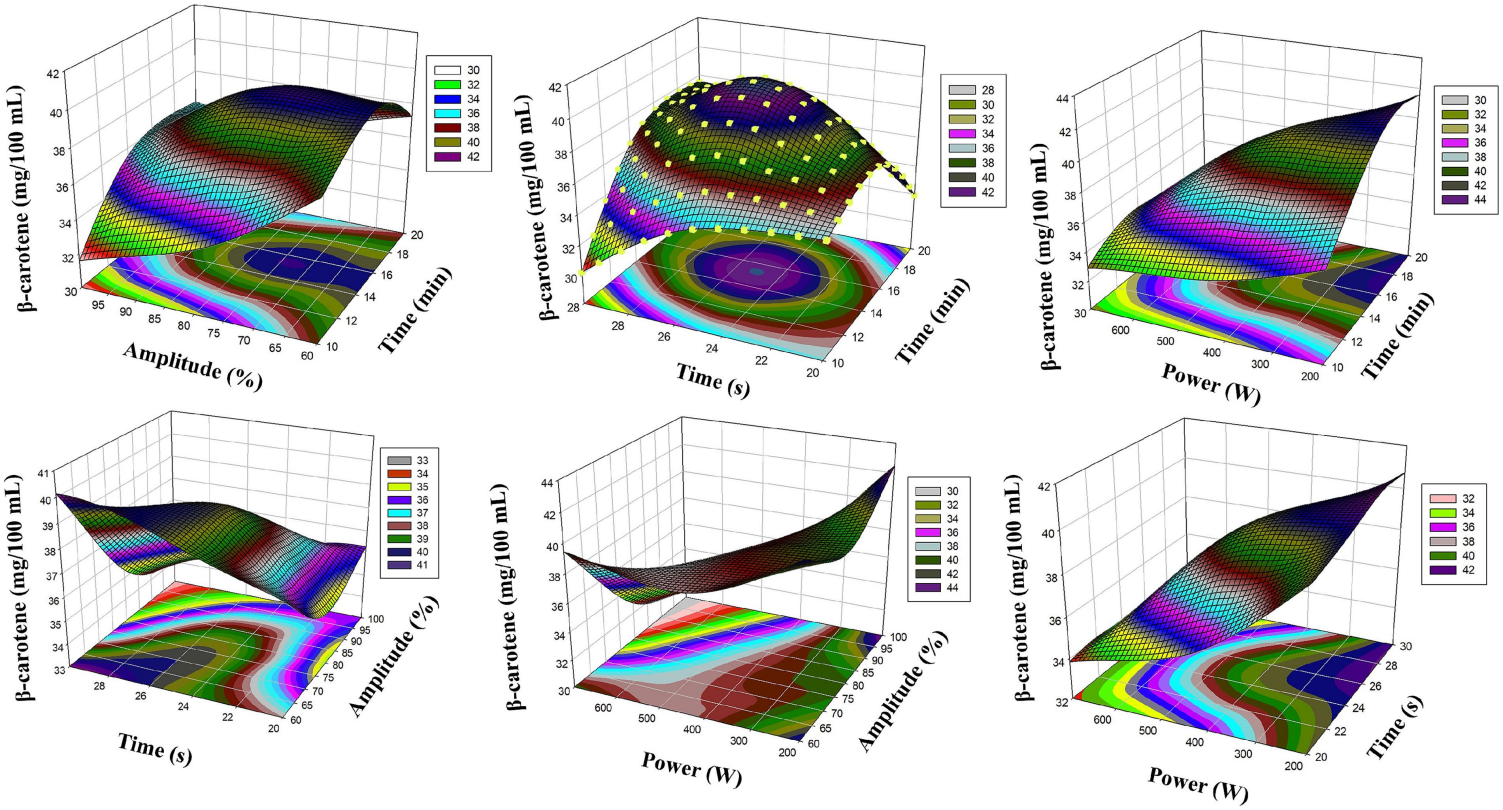
Reaction surface plots of *β*-carotene (mg/100 mL) as a function of significant interaction (3D) (RSM).

Figure 2 presents the 3D reaction surface plots of *β*-carotene level obtained by RSM model. In particular, the interaction between ultrasound amplitude (X_2_) and microwave power (X_4_) caused a significant increase in β-carotene content. It was observed that β-carotene concentration increased significantly when the amplitude was increased to 75% levels and microwave power was kept in the range of 250–450 W. This shows that pigment structures were released more effectively due to the synergistic effect of amplitude and power parameters. However, the interaction between ultrasound duration (X_1_) and microwave duration (X_3_) on *β*-carotene levels is also remarkable. As seen in Figure 2, when ultrasound applications in the range of 15–20 min were combined with microwave duration of 25 s, maximum values were reached in *β*-carotene amount. This finding shows that both short-term thermal effect was avoided and structure deterioration was prevented and cell wall permeability reached maximum. In this context, it is understood that both acoustic cavitation and rapid microwave heating play an important role in *β*-carotene extraction.

**Figure 3:**
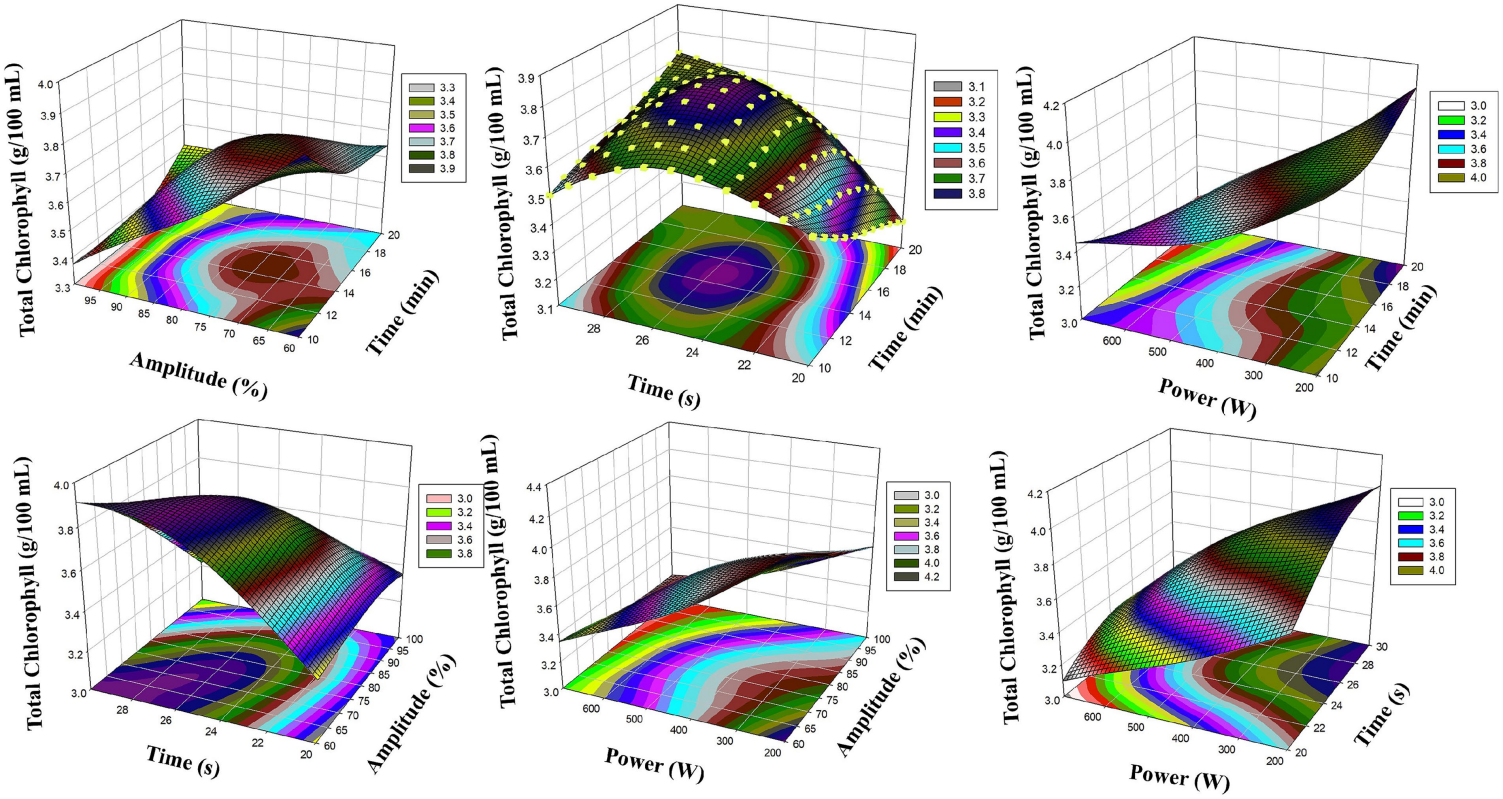
Reaction surface plots of Total Chlorophyll (g/100 mL) as a function of significant interaction (3D) (RSM).

Figure 3 shows the RSM surface graphics of total chlorophyll levels. In particular, the interaction between ultrasound amplitude (X_2_) and microwave power (X_4_) provided a significant increase in chlorophyll content. The cavitation intensity increased with the increase in amplitude, which facilitated the passage of chlorophyll out of the cell. The application of microwave power at controlled levels (200–300 W) supported the extraction of the pigment without thermal degradation. In addition, the smooth slope formed on the surfaces between X_1_ and X_2_ revealed that these two parameters had a parallel effect on chlorophyll extraction. As a result, RSM analyses graphically support optimization and clearly show the interaction of process parameters with each other.

### 3.2 Evaluation of results obtained with MLP

In this study, Multilayer Perceptron (MLP), an artificial neural network based model, was used to estimate *β*-carotene and total chlorophyll levels and gave very successful results. In order to prevent the possible overfitting problem, the results of a tenfold crossover procedure applied in the model were evaluated and to avoid this situation where the test data was highly inaccurate while the prediction of the training data was successfully, the data was divided as 80% training and 20% testing in the *β*-carotene prediction, while the data was divided as 90% training and %10 testing in the total chlorophyll prediction. Under optimum conditions, the experimentally obtained *β*-carotene value was 42.85 ± 2.45 mg/100 mL, while the MLP estimated value was 42.20 mg/100 mL. Similarly, the experimental result for total chlorophyll was 4.42 ± 0.14 mg/100 mL, while the MLP estimate was 4.26 mg/100 mL. These values show extremely low differences with deviations of 1.52 and 3.62%, respectively, revealing that MLP provides reliable outputs close to the real values. The structure of the MLP model is presented in Figure 1.

Prediction results obtained with the MLP model showed high agreement with experimental trends for both *β*-carotene and chlorophyll. This fit arises from the capacity of MLP to effectively learn nonlinear relationships between variables. In particular, for *β*-carotene predictions, MLP appears to produce predictions with similar accuracy to RSM across all trials. Although the prediction differences are slightly higher for total chlorophyll because it is a more sensitive compound to environmental factors, the model success is generally satisfactory.

**Figure 4:**
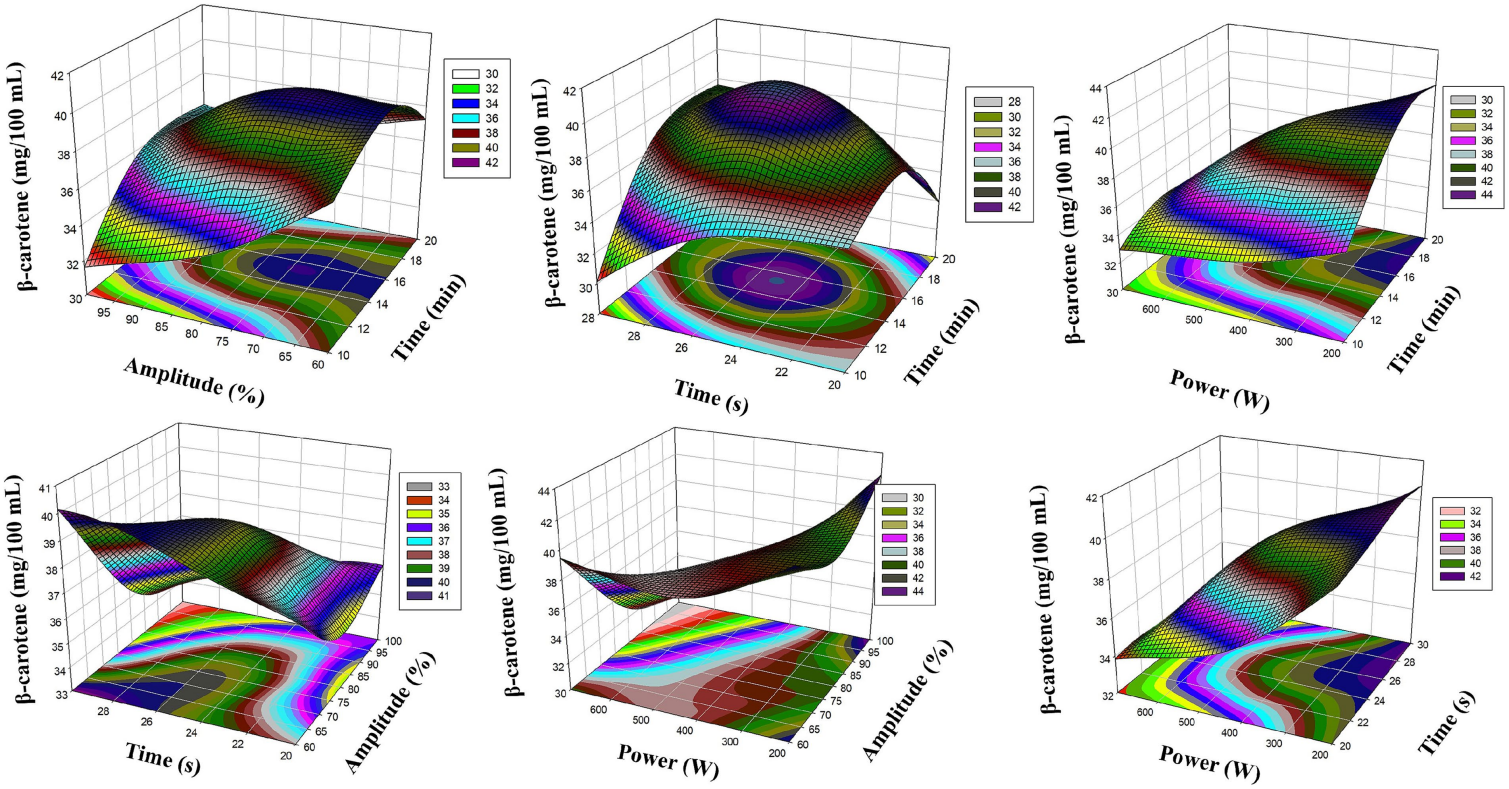
Reaction surface plots of *β*-carotene (mg/100 mL) as a function of significant interaction (3D) (MLP).

**Figure 5:**
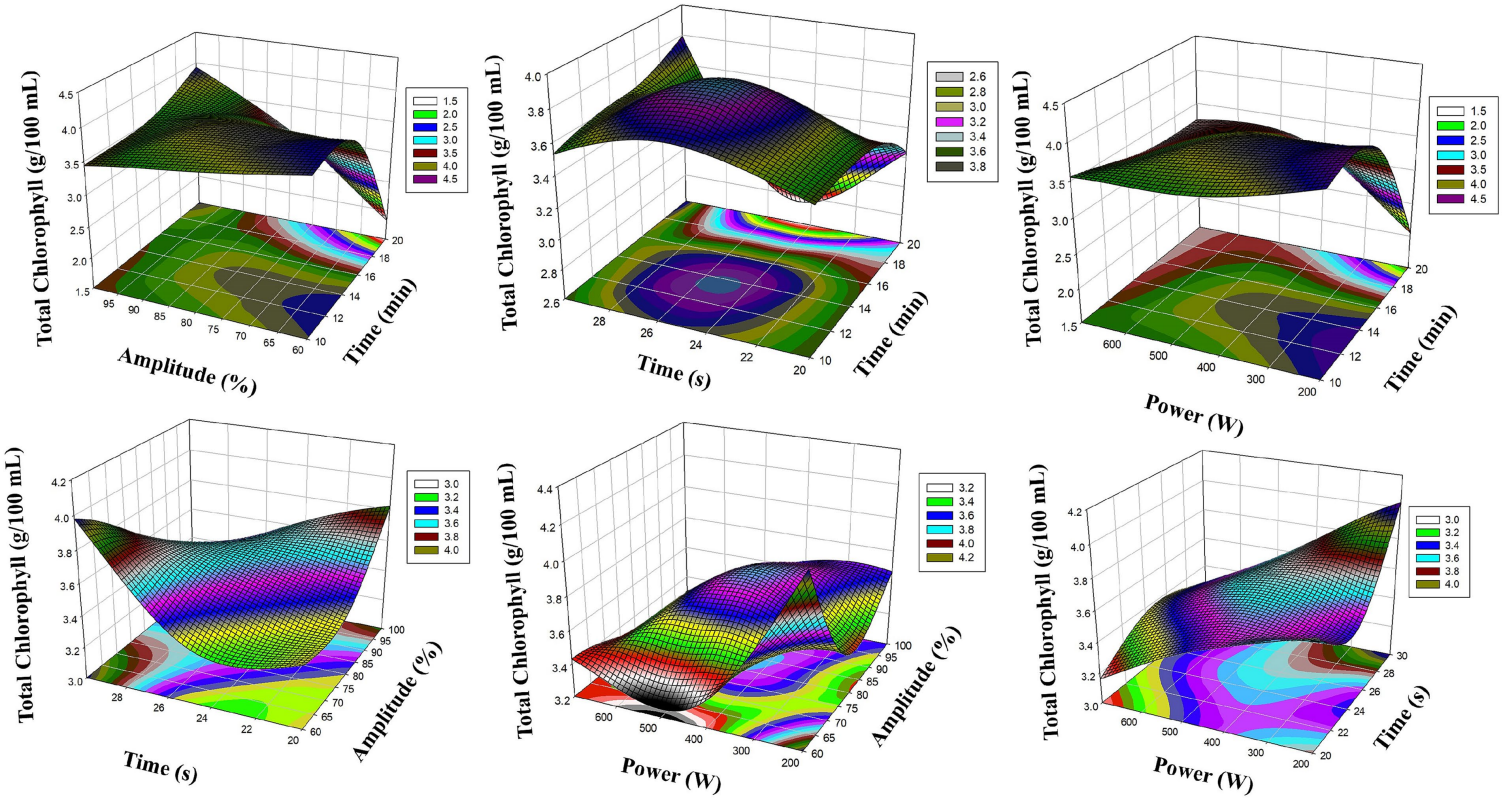
Reaction surface plots of Total Chlorophyll (g/100 mL) as a function of significant interaction (3D) (MLP).

Looking at the graphical analysis, Figures 4, 5 present the 3D surface graphics of the MLP model and successfully reflect the experimental trends. Figure 1 shows that *β*-carotene levels reach their maximum especially in combinations of ultrasound amplitude (X_2_) and microwave power (X_4_). Similarly, the synergistic effects of X_1_ (ultrasound duration) and X_2_ (amplitude) parameters on total chlorophyll levels are clearly reflected in Figure 4. The surface slopes in the graphs show that the MLP model can accurately capture not only the fundamental relationships but also the complex interactions.

When evaluated in general, the MLP model stands out as a forecasting tool that works with high accuracy both graphically and numerically. MLP, which has a more flexible structure compared to RSM, becomes more powerful especially as the volume of the data set increases. In this study, the fact that MLP gives outputs that are quite compatible with RSM shows its usability in multivariate optimization studies in the field of food processing. In addition, MLP’s ability to better represent multidimensional and nonlinear systems compared to classical linear models makes it a powerful alternative for next-generation decision support and process development tools.

### 3.3 RSM and MPL comparison

In this study, both the RSM, a classical modeling method, and the MLP, an artificial intelligence-based method, were used to estimate *β*-carotene and total chlorophyll contents. Both methods provided high accuracy and produced estimates close to the experimental results. According to the RSM model, a deviation of 4.11% was obtained in β-carotene estimation and 3.39% in total chlorophyll estimation, while in the MLP model, these differences were calculated as 1.52 and 3.62%, respectively. Numerically, it is seen that MLP demonstrates a stronger prediction performance with a lower deviation rate, especially in terms of β-carotene.

The RSM model enables statistical optimization of experimental design while clearly defining the relationships between dependent and independent variables through the regression equations it generates. In this respect, it offers advantages in interpreting variable effects and examining their significance. However, RSM may be limited in systems with complex and non-linear relationships. In this context, the MLP model offers a more flexible structure thanks to the learning ability of artificial neural networks and can more successfully represent non-linear interactions between multiple parameters. When evaluated graphically, the 3D surface graphs created by both models show similar trends. In both RSM and MLP models, the interaction between amplitude (X_2_) and microwave power (X_4_) for *β*-carotene is reflected in a distinct increase curve. For total chlorophyll, the interaction between X_1_ (ultrasound duration) and X_2_ (amplitude) has a more dominant effect in both models. However, MLP graphs have shown the possible synergistic interactions in the system more clearly, especially by presenting complex surface shapes and curves in a more detailed and smooth manner. This highlights the high data processing capacity and sensitivity to detail of MLP.

As presented in the Table 3, *R*^2^, RMSE (Root Mean Square Error) and MAPE (Mean Absolute Percentage Error) criteria [adopted from (17)] were used to test the accuracy of the prediction models and to compare the success of RSM and MLP methods.

**Table 3:** Model accuracy comparison criteria.

Parameter	Equation	Equation number	Desirable value
*R*^2^	$=1-\frac{\sum_{i=1}^{n}\;{\left(z-z\prime \right)}^{2}}{\sum_{i=1}^{n}z_{i}^{2}}\;$	(4)	Close to 1
RMSE	= $\sqrt{\frac{\sum_{i=1}^{n}{\left(z-z\prime \right)}^{2}}{N}}$	(5)	Close to 0
MAPE	$=\frac{1}{N}\sum_{i=1}^{n}|\frac{z-z\prime }{z}|$	(6)	Close to 0

*R*^2^: R-squared; RMSE: Root Mean Square Error; MAPE: Mean Absolute Percentage Error; z = experimental value, z′ = predicted value.

In conclusion, while both models have strengths in terms of optimization and prediction, considering the complexity and multivariate structure of the data set, it can be said that the MLP model is more flexible and effective. While the RSM model offers advantages in terms of interpretability, the MLP model stands out for its prediction accuracy and comprehensive structure that encompasses all system dynamics. Therefore, the combined use of RSM and MLP models in future studies could yield stronger and more balanced results from both theoretical and practical perspectives.

According to sensitivity analysis results (Table 4); the most important parameter to which the output is sensitive is Ultrasound duration (X_1_) (min). Then Microwave Power (X_4_) (W) and Microwave duration (X_3_) (s) are effective, respectively. Although the Ultrasound Amplitude (X_2_) (%) variable is effective (with a sig. Coef. of 0.0137 and a normalized sig. of 41.8%), its relative contribution to predicting the output variable (*β*-carotene (mg/100 mL)) is quite low compared to other variables.

**Table 4:** Independent variable (β-carotene) importance.

	Importance	Normalized importance (%)
Ultrasound duration (X_1_) (min)	0.328	100.00%
Ultrasound Amplitude (X_2_) (%)	0.137	41.80%
Microwave Duration (X_3_) (s)	0.247	75.40%
Microwave Power (X_4_) (W)	0.288	87.70%

Normalized importance values reflect the relative contribution of each independent variable to the MLP model’s prediction of *β*-carotene, scaled such that the highest importance = 100%.

According to sensitivity analysis results (Table 5); the most important parameters to which the output is sensitive are microwave power (X_4_) (W), microwave duration (X_3_) (s) and ultrasound amplitude (X_2_) (%) variables, respectively. Although the ultrasound duration (X_1_) (min) variable has an effect on the dependent variable (with a sig. Coef. of 0.161 and a normalized sig. of 54.4%), its relative contribution to predicting the output variable (Total Chlorophyll (g/100 mL)) is quite low compared to other variables.

**Table 5:** Independent variable (Total chlorophyll) importance.

	Importance	Normalized Importance
Ultrasound Duration (X_1_) (min)	0.161	54.4%
Ultrasound Amplitude (X_2_) (%)	0.260	87.8%
Microwave Duration (X_3_) (s)	0.284	96.3%
Microwave Power (X_4_) (W)	0.295	100.00%

Normalized importance values reflect the relative contribution of each independent variable to the MLP model’s prediction of total chlorophyll, scaled such that the highest importance = 100%.

As can be seen in the summary table (Table 6) created according to the evaluation criteria, *R*^2^ values range between 0.034 and 0.347, RMSE values range between 0.989 and 0.993 and MAPE values range between 0.0566 and 0.0922. The predictive power and accuracy of both methods used in the study are quite high. When comparing the two methods used in the study, especially considering the RMSE and MAPE values, it was determined that the MLP method was slightly more successful than RSM in predicting both independent variables (*β*-carotene (mg/100 mL) and Total Chlorophyll (g/100 mL)).

**Table 6:** Summary of benchmarking.

Parameters	β-carotene (mg/100 mL)		Total Chlorophyll (g/100 mL)	
	**RSM**	**MLP**	**RSM**	**MLP**
*R*^2^	0.993	0.993	0.989	0.990
RMSE	0.347	0.323	0.052	0.034
MAPE	0.0566	0.0566	0.0922	0.0574

RSM: response surface methodology; MLP: multilayer perceptron; R^2^: R-squared; RMSE: root mean square error; MAPE: mean absolute percentage error.

### 3.4 Bioactive compounds

**Figure 6:**
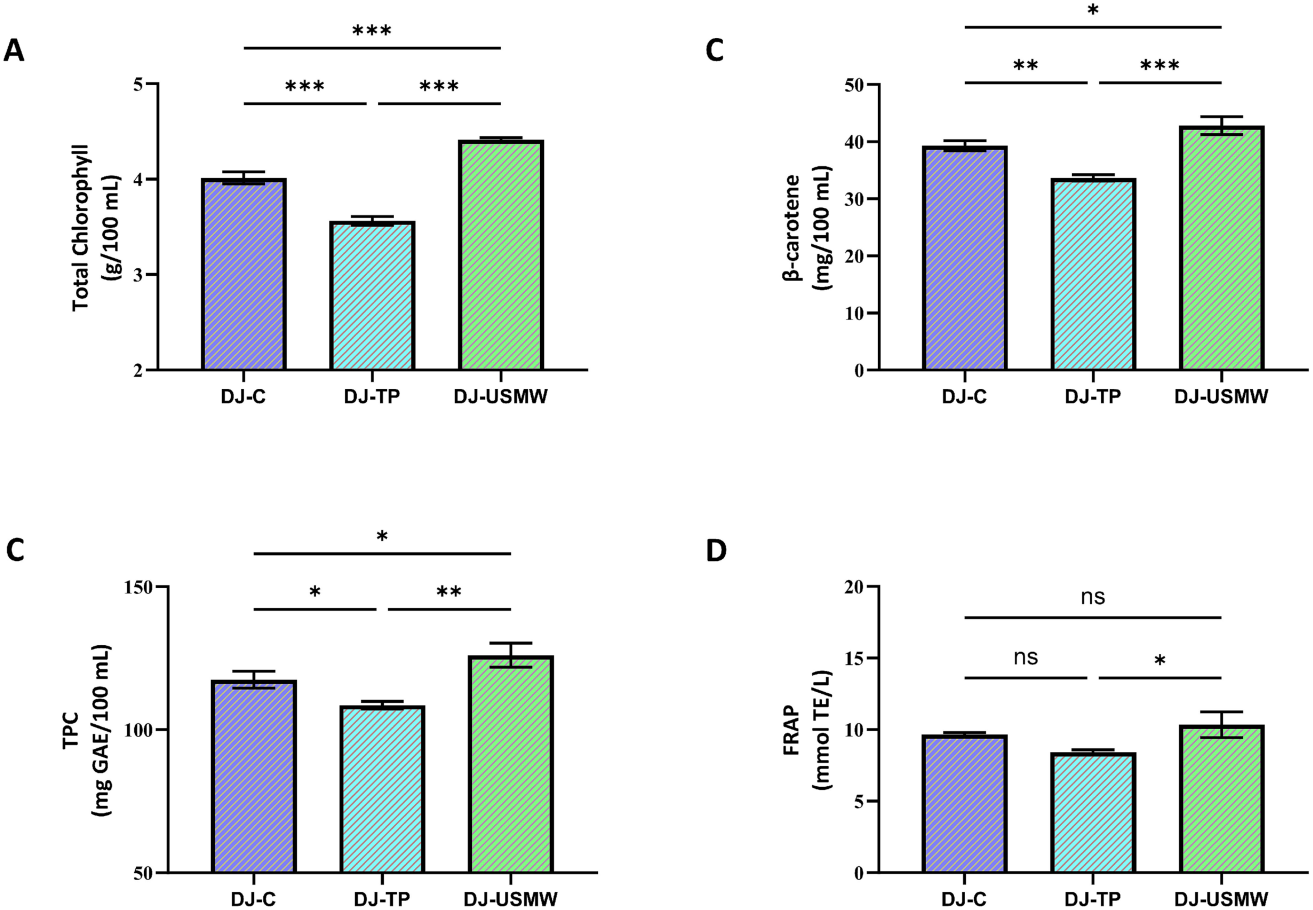
Effects of different processing methods on total chlorophyll **(A)**, β-carotene **(B)**, total phenolic content (TPC) **(C)**, and ferric reducing antioxidant power (FRAP) **(D)** of dill juice samples. DJ-C: Control (untreated), DJ-TP: Thermal pasteurized, DJ-USMW: Ultrasound + Microwave treated. Results are expressed as mean ± standard deviation (*n* = 3). *Significance levels: *p < 0.05; **p < 0.01; **p < 0.001; ns: not significant.

**Figure 7:**
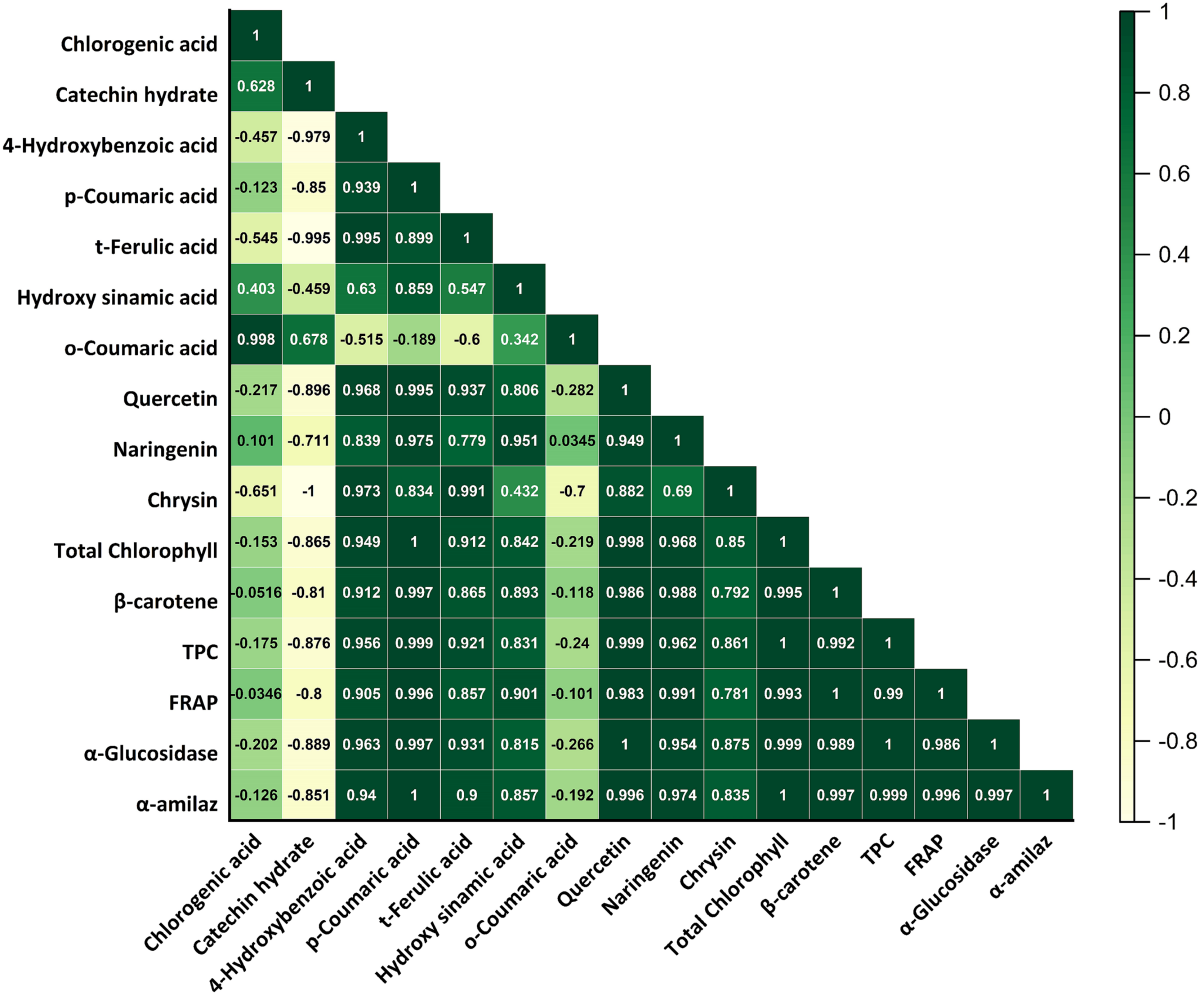
Pearson correlation matrix showing relationships among phenolic compounds, bioactive components, antioxidant capacity (FRAP), and enzyme inhibition activities (α-glucosidase and α-amylase) in dill juice samples.

The combined process of DJ-USMW achieved the highest values in all bioactive components. In particular, the total chlorophyll content in the DJ-USMW sample was determined to be 4.42 g/100 mL, which is significantly higher than the control (4.01 g/100 mL) and DJ-TP (3.56 g/100 mL) groups (*p < 0.05). The decrease observed in the DJ-TP process indicates that heat application degrades chlorophyll pigments. In a study, it was found that microwave treatment applied during the extraction of *Chlorella vulgaris* increased the total chlorophyll content (35). According to Pearson correlation analysis, a positive and significant relationship (*r* ≈ 0.88, *p* < 0.01) was found between chlorophyll and FRAP. This shows that chlorophyll contributes not only to color but also to antioxidant capacity (Figures 6A, 7).

The *β*-carotene content reached its highest level (42.82 mg/100 mL) with the DJ-USMW application. The control sample had a value of 39.30 mg/100 mL, while DJ-TP had a value of 33.65 mg/100 mL, demonstrating sensitivity to heat (**p < 0.01). Pearson correlation revealed strong positive relationships between *β*-carotene and both TPC (*r* ≈ 0.93, *p* < 0.001) and FRAP (*r* ≈ 0.95, *p* < 0.001). This finding suggests that carotenoids work in conjunction with phenolic compounds to enhance antioxidant defense and that the DJ-USMW process supports this synergy (Figures 6B, 7). Muñoz-Almagro et al. (13) emphasized that hybrid technology combining ultrasound and microwave is an effective pretreatment because it induces structural changes in molecules and facilitates subsequent enzymatic action (13).

In terms of TPC, the DJ-USMW sample stands out with a value of 126.08 mg GAE/100 mL; this value is significantly higher than the control (117.51 mg GAE/100 mL) and DJ-TP (108.65 mg GAE/100 mL) samples (**p < 0.01). This increase may be due to ultrasonic cavitation and microwave heat breaking down cell walls, thereby facilitating the release of phenolic compounds (Figure 6C). Tokatlı Demirok and Yıkmış (36) conducted a study applying ultrasound-microwave to mandarin juice. The results of this study are consistent with our study. As a result of the combined application of ultrasound and microwave to mandarin orange juice, it was observed that the TPC value of mandarin orange juice increased statistically compared to the control sample. The increase in TPC value may be due to the sudden increase in temperature and the destruction of cells caused by the ultrasound amplitude and the cavitation effect it causes (36). Phenolic compounds, which are secondary metabolites derived from plants, play an important role in the development of the color and aroma characteristics of fruit juices. In another study conducted on watermelon juice, the lowest total phenolic content was found in the untreated watermelon juice sample at 143.10 mg GAE/100 mL. The highest total phenolic content value was found to be 852.57 mg GAE/100 mL after 10 min of ultrasound and 1 min 50 s of microwave treatment (37). In a study conducted on fresh lychee juice, unlike our study, the TPC value in the untreated control sample was 90.38 ± 1.21 mg GAE/100 mL, while in the sample treated with a combination of 10 min of ultrasound and microwave, it was found to be 81.84 ± 0.72 mg GAE/100 mL. The decrease in TPC value as a result of this combined application is statistically significant (*p* < 0.05) (38). The oxidative degradation of phenolic compounds may be responsible for the decrease in TPC during sonication (39). Pearson analysis shows a very high positive correlation between TPC and FRAP (*r* ≈ 0.97, *p* < 0.001). In addition, a negative correlation (*r* ≈ −0.89, *p* < 0.01) was found between TPC and *α*-glucosidase inhibition, which supports the potential antidiabetic effect of phenolic compounds.

The FRAP value also peaked at 10.34 mmol TE/L in the DJ-USMW sample. This value is significantly higher than the control (9.63 mmol TE/L) and DJ-TP (8.43 mmol TE/L) samples (*p < 0.05). The Pearson correlation matrix shows high positive correlations between FRAP and TPC (*r* ≈ 0.97), *β*-carotene (*r* ≈ 0.95), and chlorophyll (*r* ≈ 0.88). These results clearly demonstrate that the DJ-USMW process preserves natural antioxidants such as phenolic compounds, carotenoids, and chlorophyll and enhances total antioxidant capacity through synergistic effects (Figures 6D, 7).

### 3.5 Phenolic compounds

Phenolic compounds act as effective reducing agents due to their ability to donate electrons or hydrogen atoms. This property enhances their capacity to neutralize free radicals, thereby contributing significantly to their antioxidant potential. Furthermore, they can form chelate complexes with transition metals, particularly iron and copper, thus preventing these metals from catalyzing the formation of reactive oxygen species (40). When comparing the phenolic compound contents of dill juice samples subjected to DJ-C, DJ-TP and DJ-USMW treatments, the highest total phenolic compound content was detected in the DJ-USMW sample (56.54 ± 3.77 μg/mL) (Table 7). Supplementary material illustrates the HPLC chromatograms of phenolic compounds detected in control, thermal pasteurized, and ultrasound–microwave treated dill juice samples. This value is significantly higher than that of the control (37.78 ± 1.41 μg/mL) and DJ-TP (32.77 ± 2.42 μg/mL) samples (*p* < 0.05). This result indicates that combined non-thermal processes increase the extraction of phenolic compounds by disrupting cell structure. In another study on kiwi juice, similar to our study, it was found that the phenolic compound content of kiwi juice increased significantly with ultrasonic treatment (41). The phenomenon of cavitation, which destroys fruit juice cells and releases intracellular components, is responsible for this increase (42). According to Pearson correlation analysis, a high positive correlation was determined between the total phenolic compound content and FRAP (*r* = 0.921) and *α*-glucosidase inhibition (*r* = 0.885) (Figure 7). These findings confirm that phenolic compounds are strongly related to antioxidant capacity and enzyme inhibition activity.

**Table 7:** Phenolic compounds analysis results of DJ-C, DJ-TP and DJ-USMW.

Phenolic compound (μg/mL)	DJ-C (Control)	DJ-TP (Thermal Pasteurized)	DJ-USMW (Ultrasound + Microwave)
Chlorogenic acid	33.01 ± 0.78^a^	30.56 ± 2.52^a^	29.96 ± 0.54^a^
Catechin hydrate	0.55 ± 0.03^b^	0.56 ± 0.06^b^	0.27 ± 0.08^a^
4-Hydroxybenzoic acid	0.50 ± 0.04^b^	0.00 ± 0.00^a^	2.05 ± 0.15^c^
p-Coumaric acid	0.44 ± 0.04^b^	0.00 ± 0.00^a^	0.8 ± 0.09^c^
t-Ferulic acid	1.03 ± 0.16^a^	0.19 ± 0.06^a^	6.05 ± 0.54^b^
Hydroxycinnamic acid	0.59 ± 0.04^b^	0.00 ± 0.00^a^	0.54 ± 0.04^b^
o-Coumaric acid	0.06 ± 0.03^a^	0.02 ± 0.00a	0.00 ± 0.00^a^
Quercetin	1.31 ± 0.21^a^	1.27 ± 0.08^a^	1.35 ± 0.10^a^
Naringenin	0.31 ± 0.09^a^	0.18 ± 0.02^a^	0.35 ± 0.05^a^
Chrysin	0.00 ± 0.00^a^	0.00 ± 0.00^a^	15.19 ± 3.90^b^
Total	37.78 ± 1.41^a^	32.77 ± 2.42^a^	56.54 ± 3.77^b^

The results are the mean ± standard deviation (*n* = 3). The values marked with different letters within the line are significantly different from each other (*p* < 0.05). DJ-C: Control dill juice; DJ-TP: Thermal pasteurized dill juice; DJ-USMW: Ultrasound + Microwave dill juice.

Significant differences were observed between groups in some individual phenolic compounds. In particular, the t-ferulic acid content was significantly higher in the DJ-USMW sample at 6.05 ± 0.54 μg/mL, while it was measured at 1.03 ± 0.16 μg/mL in DJ-C and only 0.19 ± 0.06 μg/mL in DJ-TP (Table 7). 4-hydroxybenzoic acid and p-coumaric acid were only detected in the DJ-USMW group at levels of 2.05 ± 0.15 and 0.80 ± 0.09 μg/mL, respectively. It is understood that DJ-TP completely degrades these compounds. In another study conducted on mandarin juice, it was observed that the thermal pasteurisation process reduced the total phenolic compound content in mandarin juice (43). In a study conducted on Brzezina blackberry pomace extracts, similar to our study, it was observed that the p-coumaric acid value increased as a result of ultrasound and microwave applications (44). According to the Pearson correlation matrix, p-coumaric acid is highly positively correlated with *α*-amylase inhibition activity (*r* = 0.812) (Figure 7). These results show that the DJ-USMW process not only provides phenolic richness but also significantly improves biological activity.

Another notable aspect of the DJ-USMW application is the chrysin compound, which was detected exclusively in this group. Chrysin, measured at 15.19 ± 3.90 μg/mL in the DJ-USMW group, was not detected in the other two samples (Table 7). This indicates that the synergistic effect of ultrasound and microwave can release phenolic compounds that were previously unbound within the cells. According to Pearson analysis, a very high correlation was found between chrysin and *α*-glucosidase inhibition activity (*r* = 0.894) (Figure 7). This finding suggests that this compound may play an important role in antidiabetic effects. In contrast, no significant differences were observed between the three groups in some compounds such as chlorogenic acid, quercetin and naringin (*p* > 0.05). For example, chlorogenic acid levels were measured as 33.01 ± 0.78, 30.56 ± 2.52, and 29.96 ± 0.54 μg/mL in DJ-C, DJ-TP, and DJ-USMW, respectively, with no statistical difference between the groups (Table 7). This indicates that these compounds are structurally more resistant to the applied treatments. However, a moderate positive correlation (r = 0.674) was observed between chlorogenic acid and FRAP value (Figure 7). Overall, it can be concluded that the DJ-USMW process both increases the total phenolic compound content and enhances the biofunctional effects of these compounds.

### 3.6 Antidiabetic activity

**Figure 8:**
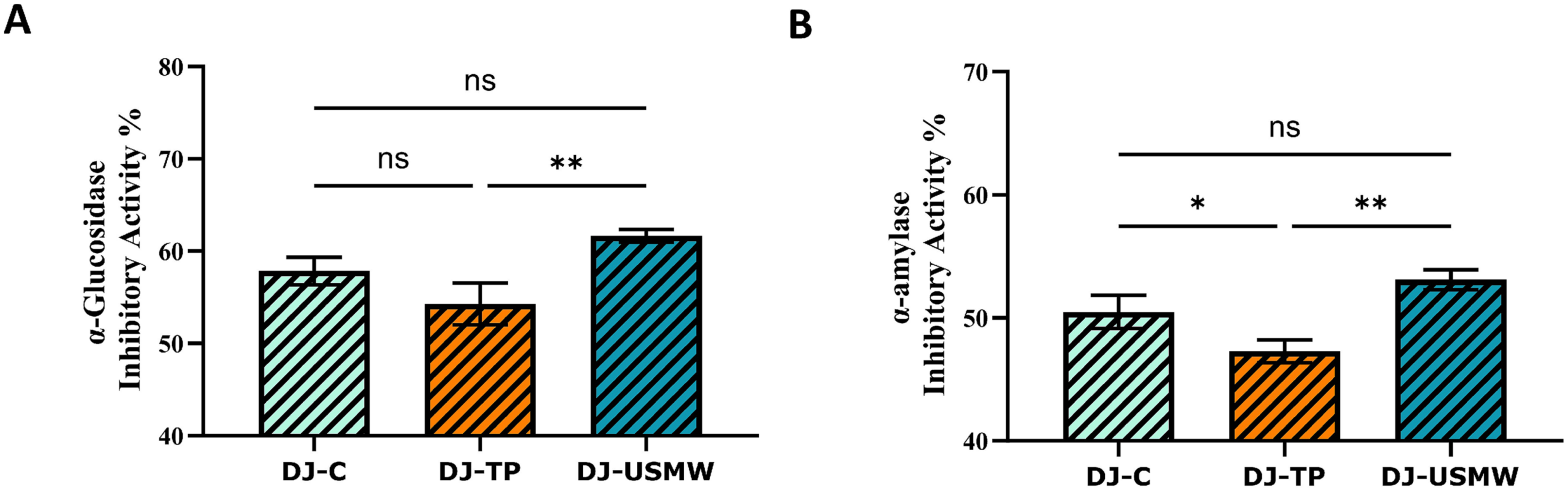
Inhibitory activity (%) of *α*-glucosidase **(A)** and *α*-amylase **(B)** enzymes by differently processed dill juice samples (DJ-C: Control, DJ-TP: Thermal Pasteurized, DJ-USMW: Ultrasound + Microwave). Data are presented as mean ± standard deviation (*n* = 3). Statistical significance was evaluated using one-way ANOVA followed by Tukey’s post-hoc test (*p < 0.05, *p < 0.01, ns: not significant).

The inhibitory effects of dill juice on *α*-glucosidase and α-amylase enzymes showed significant differences depending on the type of treatment applied. In the inhibitory activity results evaluated for the α-glucosidase enzyme (Figure 8A), the DJ-C sample in the control group showed approximately 57.84% inhibition. In the DJ-TP sample subjected to thermal pasteurisation, this rate decreased to 54.27%, and the difference between the two was found to be statistically insignificant (ns). In contrast, the DJ-USMW sample treated with the optimized combination of ultrasound and microwave reached an inhibition rate of 61.65%, which was significantly higher than that of the DJ-TP sample (**p < 0.01). This increase indicates that the USMW process positively contributes to the preservation or release of α-glucosidase-inhibiting phenolic compounds. Yıkmış et al. (45) reported that pomegranate juice treated with a combined ultrasound-microwave application resulted in the highest α-glucosidase enzyme inhibition in the treated pomegranate juice (TS-PS) samples. The results of this combined application study are consistent with our study (45). In another study, a functional sugar-free beverage was produced using gilaburu fruit and products derived from it. According to the study’s findings, the microwave-assisted water extract of the gilaburu fruit inhibited *α*-glucosidase enzyme activity by 54%, while the functional sugar-free beverage inhibited α-glucosidase enzyme activity by 77%. This effect is supported by the methods used, which increase the phenolic compound content of the gilaburu fruit, thereby enhancing its antidiabetic potential (46).

Similarly, inhibitory effects on the α-amylase enzyme also varied depending on the process (Figure 8B). The lowest inhibition rate (47.28%) was observed in the thermally pasteurized DJ-TP sample, while this rate was measured as 50.49% in the sample DJ-C, showing a significant difference compared to DJ-TP (*p < 0.05). In the DJ-USMW group, the highest α-amylase inhibitory activity was determined to be 53.11%, which was significantly higher than the other two groups (**p < 0.01). This confirms that the USMW process is a more effective method for enzyme inhibition. Yıkmış et al. (45) in their pomegranate juice study, the highest inhibition was observed on α-amylase in the TS-PJ sample, similar to α-glucosidase enzyme inhibition (45). In another study evaluating the antidiabetic potential of asparagus water, it was found that ultrasonic treatment significantly increased α-amylase inhibitory activity by 25.61% compared to the untreated group (47). The studies conducted and reviewed show that ultrasound alone or in combination with microwaves increases the antidiabetic potential, supporting the results of our study.

The results obtained show that the DJ-TP tends to reduce both α-glucosidase and α-amylase inhibitory activities, whereas the USMW process provides a significant advantage by increasing these activities. The synergistic effect created by the combination of ultrasound and microwave can facilitate the extraction of phytochemical components, enabling higher yields of phenolic compounds, flavonoids, or saponins. Additionally, the preservation of heat-sensitive bioactive compounds without degradation may also be a source of this effect. A study conducted on a melon and sugarcane juice mixture using the combination of thermosonication and microwave highlights that the applied method is an effective alternative processing method that enhances physicochemical and nutritional quality, preserves bioactive components, and extends shelf life, similar to our study (48). When examining the effects of different application techniques on the chemical composition of aronia juice, it was emphasized that ultrasonic and microwave-assisted processes in particular caused significant increases in total sugar and organic acid content. At the same time, they also led to noticeable and positive changes in sensory properties. Basically, modern extraction techniques appear to be advantageous methods in terms of efficiency and sensory appeal (49).

As a result, it has been demonstrated that dill juice obtained through the USMW process has significant antidiabetic potential by effectively inhibiting α-glucosidase and α-amylase enzymes. In this context, USMW applications can be considered a more effective and sustainable technology in the functional beverage development process as an alternative to traditional thermal processes. According to Pearson correlation analysis, strong positive relationships were found between inhibitory activities and total phenolic compounds, flavonoids, and antioxidant capacity (Figure 7). This supports the role of phenolic structures in enzyme inhibition.

### 3.7 Bioavailability

Data obtained at the beginning of simulated digestion processes, i.e., in the ‘undigested’ phase, show that the applied treatments have significant effects on the bioactive components of dill juice. The DJ-USMW group yielded significantly higher results than the DJ-C and DJ-TP groups in all parameters (chlorophyll, *β*-carotene, TPC, and FRAP value) (*p* < 0.05). For example, total chlorophyll was 4.42 g/100 mL in the DJ-USMW sample, while this value was 4.01 g/100 mL in DJ-C and 3.56 g/100 mL in DJ-TP. Similarly, β-carotene content was also highest in DJ-USMW at 42.82 mg/100 mL. These findings demonstrate that non-thermal technologies are effective in preserving thermolabile (heat-sensitive) components. In contrast, DJ-TP degrades bioactive components, reducing their quantities. A study conducted on cranberry bush fruit revealed that the ultrasound-microwave-assisted extraction (UMAE) method is the most efficient method for extracting phenolic compounds from cranberry fruit. UMAE provided the highest TPC, antioxidant capacity, and higher epicatechin levels compared to other methods (50).

In the oral phase of digestion, a decrease in bioactive components was observed in all groups, but the DJ-USMW sample remained statistically superior to the other groups (*p* < 0.05). For example, while the chlorophyll content in the DJ-USMW group was 3.30 g/100 mL and the *β*-carotene content was 30.27 mg/100 mL, these values were measured as 2.97 mg/100 mL and 27.64 mg/100 mL, respectively, in the DJ-C group and 2.49 mg/100 mL and 23.11 mg/100 mL in the DJ-TP group (Table 8). Although the pH and enzymatic activity of the oral environment are limited, the degradation process that begins in this phase may reduce the solubility of some compounds. However, the DJ-USMW application may have contributed to the better release of these compounds into the digestive environment by making the matrix structure more permeable. A similar trend was observed in the TPC and FRAP results; the DJ-USMW sample retained its antioxidant capacity relatively better in the oral phase. In the study of fruit juice obtained from passion fruit from different regions, after *in vitro* digestion, significant increases in TPC and total flavonoid (TFC) content were observed as a result of the ultrasonic treatment applied without any comparison between phases. The instability of vitamin C during digestion increased its loss. On the other hand, since the fruits were obtained from different regions, the antioxidant capacity of some fruits increased while that of others decreased (51). This situation shows that the geographical conditions of the content used are also an important factor.

**Table 8:** Changes in total chlorophyll, β-carotene, total phenolic content (TPC), and ferric reducing antioxidant power (FRAP) during simulated gastrointestinal digestion of dill juice samples subjected to different treatments.

Phases	Samples	Total chlorophyll (g/100 mL)	β-carotene (mg/100 mL)	TPC (mg GAE/100 mL)	FRAP (mmol TE/L)
Undigested	DJ-C	4.01 ± 0.06^b^	39.30 ± 0.89^b^	117.51 ± 2.93^b^	9.63 ± 0.15^ab^
	DJ-TP	3.56 ± 0.05^a^	33.65 ± 0.56^a^	108.65 ± 1.32^a^	8.43 ± 0.17^a^
	DJ-USMW	4.42 ± 0.02^c^	42.82 ± 1.57^c^	126.08 ± 4.18^c^	10.34 ± 0.90^b^
Oral digestion	DJ-C	2.97 ± 0.05^b^	27.64 ± 0.66^b^	83.43 ± 2.08^b^	7.23 ± 0.12^b^
	DJ-TP	2.49 ± 0.03^a^	23.11 ± 0.55^a^	74.61 ± 2.04^a^	6.32 ± 0.13^a^
	DJ-USMW	3.30 ± 0.06^c^	30.27 ± 1.60^c^	90.38 ± 4.26^b^	7.84 ± 0.52^b^
Gastric digestion	DJ-C	1.28 ± 0.02^b^	11.79 ± 0.21^b^	48.39 ± 1.20^b^	3.03 ± 0.05^b^
	DJ-TP	1.05 ± 0.04^a^	9.94 ± 0.24^a^	41.28 ± 1.10^a^	2.66 ± 0.05^a^
	DJ-USMW	1.42 ± 0.03^c^	13.02 ± 0.68^c^	52.42 ± 2.47^b^	3.24 ± 0.18^b^
Intestinal digestion	DJ-C	0.66 ± 0.02^b^	6.13 ± 0.12^b^	27.58 ± 0.69^b^	1.37 ± 0.03^b^
	DJ-TP	0.50 ± 0.03^a^	4.80 ± 0.17^a^	23.53 ± 0.63^a^	1.12 ± 0.08^a^
	DJ-USMW	0.76 ± 0.03^c^	6.38 ± 0.34^b^	30.91 ± 1.39^c^	1.47 ± 0.07^b^
Recovery %	DJ-C	16.44 ± 0.18^b^	15.61 ± 0.30^b^	23.47 ± 0.00^b^	14.18 ± 0.00^a^
	DJ-TP	14.02 ± 0.65^a^	14.27 ± 0.46^a^	21.66 ± 0.32^a^	13.34 ± 0.80^a^
	DJ-USMW	17.12 ± 0.64^b^	14.89 ± 0.24^ab^	24.52 ± 0.91^b^	14.25 ± 0.79^a^

DJ-C (Control), DJ-TP (Thermal Pasteurized), and DJ-USMW (Ultrasound-Microwave). Values are expressed as mean ± standard deviation (*n* = 3). Different superscript letters within the same digestion phase indicate significant differences between treatments (*p* < 0.05).

The stomach phase is one of the stages where bioactive components are most affected due to lower pH and more intense enzymatic activity. Significant decreases have been recorded in all parameters in this phase. However, the DJ-USMW group yielded higher results compared to the DJ-C and DJ-TP groups in terms of chlorophyll (1.42 g/100 mL), β-carotene (13.02 mg/100 mL), TPC (52.42 mg GAE/100 mL), and FRAP (3.24 mmol TE/L) values, yielding higher results compared to the DJ-C and DJ-TP groups (*p* < 0.05). In particular, these values were observed as 1.05 g/100 mL, 9.94 mg/100 mL, 41.28 mg GAE/100 mL, and 2.66 mmol TE/L, respectively, in the thermally treated DJ-TP sample, suggesting that conventional thermal processing negatively affects the stability of bioactive components under stomach conditions. In the DJ-USMW process, ultrasound may increase content release by breaking down cell walls, while microwaves may have a protective effect through homogeneous heat distribution, explaining why components remain more stable in the stomach phase.

The small intestine phase is the critical stage where bioactive compounds are absorbed. Although values continued to decrease in all samples during this phase, the DJ-USMW sample again achieved the highest values. For example, TPC was 30.91 mg GAE/100 mL in DJ-USMW, 27.58 mg GAE/100 mL in DJ-C, and only 23.53 mg GAE/100 mL in DJ-TP. The FRAP value also remained at the highest level in the DJ-USMW sample at 1.47 mmol TE/L. These differences indicate the potential of the DJ-USMW application to enhance the bioavailability of the compounds. Recovery (%) analyses also support this finding; the post-digestion recovery rate of TPC in the DJ-USMW group was 24.52%, significantly higher than the others (*p* < 0.05). This clearly demonstrates that combined non-thermal technologies are effective in increasing bioavailability by preventing the degradation of bioactive compounds during digestion. Fermented mango juice subjected to ultraviolet-assisted ultrasonic pretreatment was found to contain higher levels of bioactive components such as ascorbic acid (vitamin C), phenolic compounds, and carotenoids compared to traditional methods. It has also been emphasized that a significant portion of these compounds are released at the end of the digestive process and converted into a form that can be utilized by the body. However, under the pH conditions of the intestinal environment, the conversion of ascorbic acid into its ascorbate form results in higher vitamin C loss in the intestinal phase compared to the gastric phase (52). This situation shows that the intestinal phase is risky in terms of the absorption of bioactive components, as in our study. According to the review by Meena et al. (53), bioactive compounds must be separated from the food matrix and integrated in order to be effectively released in the digestive system. Therefore, unlike traditional thermal processes, non-thermal technologies such as ultrasonic processing stand out in this regard, particularly by breaking down cell walls in the food matrix, releasing encapsulated bioactive compounds, and making them more accessible to digestive enzymes. Additionally, their potential to increase the bioavailability of bioactive compounds supports the findings of our study (53).

### 3.8 Distinguishing processing effects using PCA and cluster analysis

**Figure 9:**
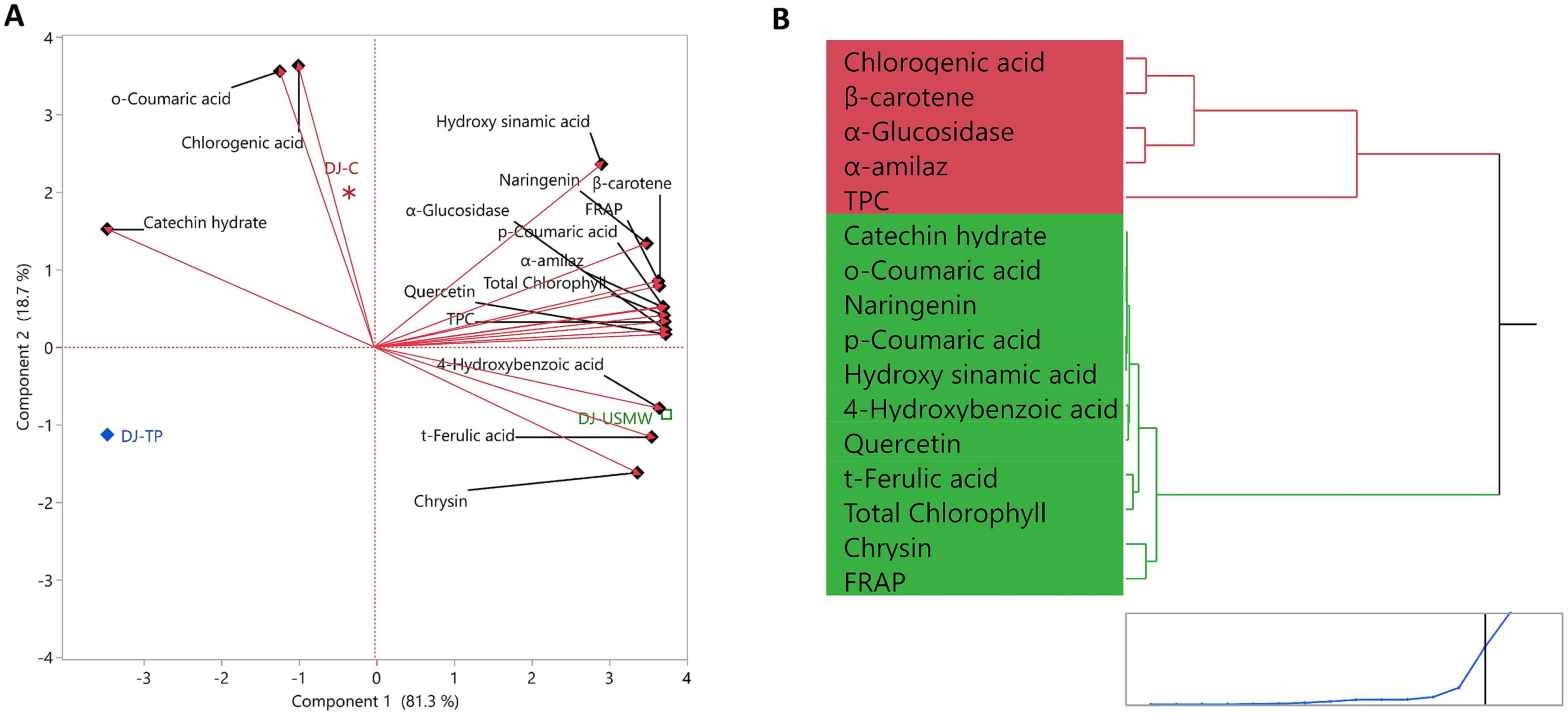
Principal component analysis (PCA) biplot **(A)** and hierarchical clustering dendrogram **(B)** illustrating the distribution and grouping of the samples based on their bioactive compound profiles. Sample codes: DJ-C – Dill Juice Control (untreated); DJ-TP – Dill Juice Thermal Pasteurized; DJ-USMW – Dill Juice Ultrasound + Microwave Treated.

The PCA biplot presented in Figure 9A clearly shows the effects of different processing techniques on the bioactive compound profile of dill juice. The two main components (PC1 and PC2) explain 81.29 and 18.71% of the total variance, respectively, representing 100% of the data. This high explanation ratio indicates that the model has strong discriminative power and meaningfully represents the samples in the dataset. The DJ-USMW sample is located in the positive PC1 and PC2 region of the PCA plane, strongly separated on the axis where parameters such as FRAP, total chlorophyll, *β*-carotene, chrysin, and *α*-glucosidase inhibition are high. These positive loading directions indicate that DJ-USMW exhibits a functionally enriched profile. On the other hand, the DJ-TP sample is located in the negative PC1 and PC2 regions, exhibiting a weak profile in terms of both phenolic compounds and color components. The DJ-C sample, on the other hand, shows a more central distribution, positioned in a positive relationship with parameters such as chlorogenic acid and total phenolic content.

The hierarchical clustering dendrogram shown in Figure 9B supports the PCA results and shows that the DJ-USMW sample is separated from the others in a single branch. This structure clearly indicates that the combined process has a statistically significant and differentiating effect on the bioactive compound profile. The DJ-C and DJ-TP samples, on the other hand, are grouped under the same main branch, indicating that they have a similar chemical composition. The fact that DJ-USMW is separated as an independent cluster in the dendrogram supports its high correlation with phenolic compounds and antioxidant parameters that have positive loading in PCA. These findings demonstrate that the DJ-USMW process is the most effective technological approach not only in terms of phenolic profile but also in terms of color and functional properties. These analyses, supported by a high variance explanation rate, prove how powerful advanced sustainable processing techniques can be as a tool for optimizing the functional components of plant-based products.

## 4 Conclusion

This study showed that functional component content and bioavailability of dill juice can be increased by combined use of ultrasound and microwave technologies (USMW). USMW application provided significant increase in parameters such as total phenolic content, *β*-carotene, chlorophyll and antioxidant capacity, and also showed high inhibitory activity against α-glucosidase and α-amylase enzymes, offering potential antidiabetic effects. Bioavailability findings evaluated with simulated digestion model indicate that USMW process contributes to the preservation of heat-sensitive components and increase of absorption potential. Predictions made with RSM and MLP models confirmed the optimizability of the process and model fit. PCA and cluster analyses revealed that USMW significantly separated the samples in terms of functionality. The obtained findings show that USMW technology offers an environmentally friendly and effective alternative to traditional methods, especially in the development of functional beverages, and provides a scientific basis for innovative applications in this field. However, since *in vitro* results may not fully predict *in vivo* bioavailability due to differences in physiological conditions, further in vivo studies will be needed to confirm these findings.

## Data Availability

The raw data supporting the conclusions of this article will be made available by the authors, without undue reservation.
