# Correction: Advanced low-thermal fortification strategy for dill juice: enhanced bioaccessibility and functional properties through MLP-RSM optimization

**DOI:** 10.3389/fnut.2025.1680243

**Published:** 2025-08-20

**Authors:** Frontiers Production Office

**Affiliations:** Frontiers Media SA, Lausanne, Switzerland

**Keywords:** *Anethum graveolens*, bioavailability, MLP optimization, non-thermal technology, ultrasound and microwave

There was a mistake in Table 5 as published. The corrected data in the table was erroneously not included. The corrected table appears below.

**Table 5:** Independent variable (Total chlorophyll) importance.

	**Importance**	**Normalized Importance**
Ultrasound Duration (X_1_) (min)	0.161	54.4%
Ultrasound Amplitude (X_2_) (%)	0.260	87.8%
Microwave Duration (X_3_) (s)	0.284	96.3%
Microwave Power (X_4_) (W)	0.295	100.00%

The original version of this article has been updated.

