# Supplementary material for: Advanced low-thermal fortification strategy for dill juice: enhanced bioaccessibility and functional properties through MLP-RSM optimization
Source: Front Nutr. 2025 Aug 1;12:1650490. doi: 10.3389/fnut.2025.1650490 (PMC12354474; doi:10.3389/fnut.2025.1650490)
Supplement: Supplementary file 1 [file Data_Sheet_1.docx]

# Advanced Low-Thermal Fortification Strategy for Dill Juice: Enhanced Bioaccessibility and Functional Properties through MLP-RSM Optimization

Seydi Yıkmış^1*^, Aylin Duman Altan^2^, Selinay Demirel^3^, Melikenur Türkol^3^, Nazlı Tokatlı^4^, Nazan Tokatlı Demirok^3^, Moneera O. Aljobair^5*^, Emad Karrar^6^, Isam A. Mohamed Ahmed^7*^

^1^Department of Food Technology, Tekirdag Namık Kemal University, 59830 Tekirdag, Türkiye

^2^Department of Industrial Engineering, Tekirdag Namık Kemal University, 59860 Tekirdağ, Turkiye

^3^Nutrition and Dietetics, Faculty of Health Sciences, Tekirdag Namık Kemal University, 59030, Tekirdag, Türkiye

^4^Department of Computer Engineering, Faculty of Engineering and Natural Sciences, Istanbul Health and Technology University, 34421 Istanbul, Türkiye

^5^Department of Sports Health, College of Sports Sciences and Physical Activity, Princess Nourah bint Abdulrahman University, Riyadh, Saudi Arabia ([moaljobair@pnu.edu.sa](mailto:moaljobair@pnu.edu.sa) )

^6^Department of Plant Sciences, North Dakota State University, Fargo 58108 ND, USA ([emad.karrar@ndsu.edu](mailto:emad.karrar@ndsu.edu) )

^7^Department of Food Sciences and Nutrition, College of Food and Agricultural Sciences, King Saud University, P. O. Box 2460, Riyadh 11451, Saudi Arabia ([iali@ksu.edu.sa](mailto:iali@ksu.edu.sa) )

*****Correspondence: [syikmis@nku.edu.tr](mailto:syikmis@nku.edu.tr) (S.Y.); [iali@ksu.edu.sa](mailto:iali@ksu.edu.sa) (I. A. M. A ); [moaljobair@pnu.edu.sa](mailto:moaljobair@pnu.edu.sa) (M.O.A)


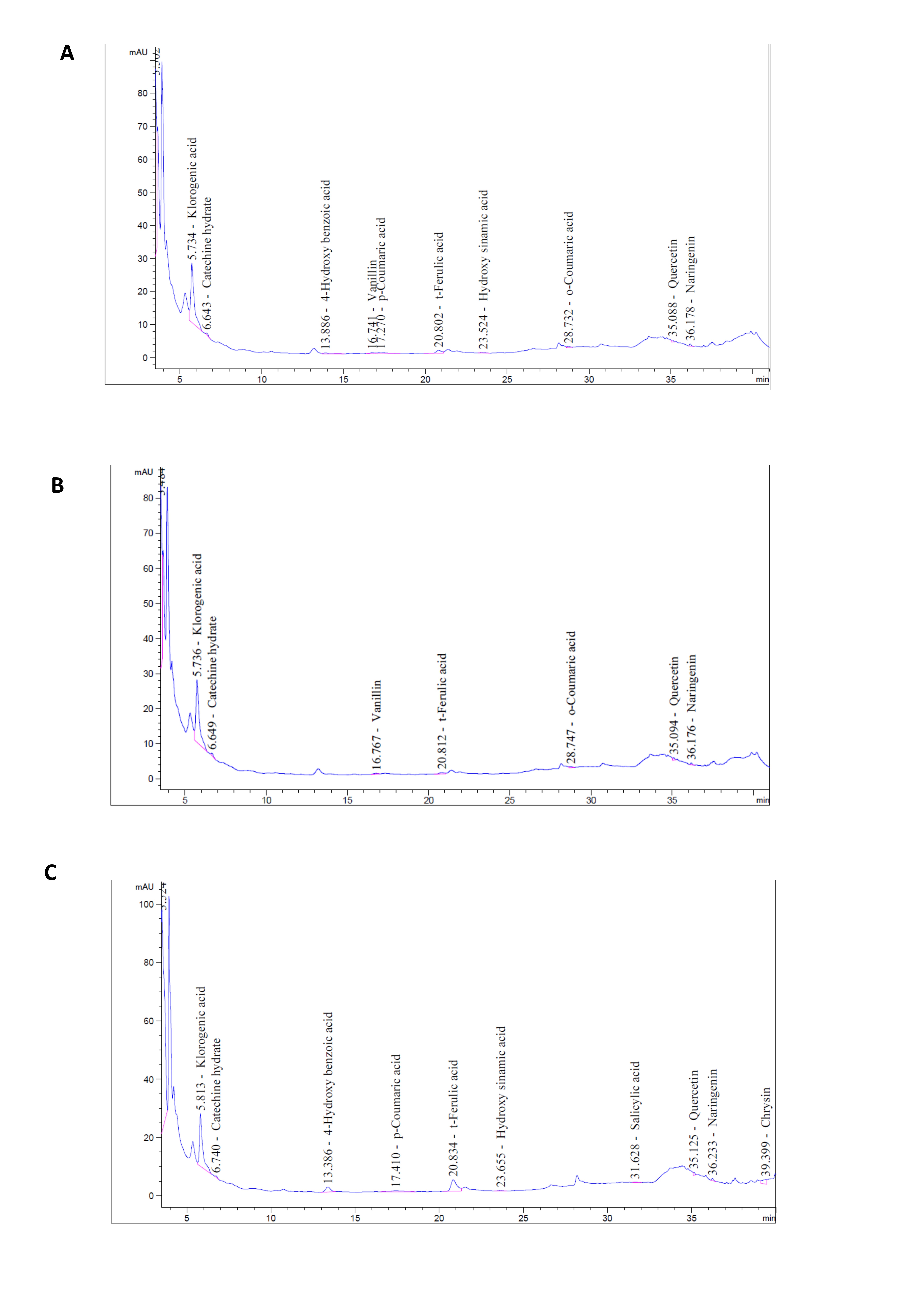


**Supplementary Figure 1.** High-performance liquid chromatography (HPLC) chromatograms of phenolic compounds identified in dill juice samples subjected to different treatments. (A) Control sample (DJ-C), (B) Thermal pasteurized sample (DJ-TP), and (C) Ultrasound + microwave treated sample (DJ-USMW). Peaks were identified based on retention time and UV–VIS spectra compared with analytical standards
